# Comparing human milk macronutrients measured using analyzers based on mid-infrared spectroscopy and ultrasound and the application of machine learning in data fitting

**DOI:** 10.1186/s12884-022-04891-w

**Published:** 2022-07-14

**Authors:** Huijuan Ruan, Qingya Tang, Yajie Zhang, Xuelin Zhao, Yi Xiang, Yi Feng, Wei Cai

**Affiliations:** 1 Department of Clinical Nutrition, Xinhua Hospital, School of Medicine, Shanghai Jiao Tong University, Shanghai, China; 2 Shanghai Key Laboratory of Pediatric Gastroenterology and Nutrition, Shanghai, China; 3 Shanghai Institute of Pediatric Research, Shanghai, China; 4 Department of Pediatric Surgery, Xinhua Hospital, School of Medicine, Shanghai Jiao Tong University, Shanghai, China

**Keywords:** Human milk analyzer, Mid-infrared spectroscopy, Ultrasound, Bland–Altman method, Machine learning

## Abstract

**Objective:**

Fat, carbohydrates (mainly lactose) and protein in breast milk all provide indispensable benefits for the growth of newborns. The only source of nutrition in early infancy is breast milk, so the energy of breast milk is also crucial to the growth of infants. Some macronutrients composition in human breast milk varies greatly, which could affect its nutritional fulfillment to preterm infant needs. Therefore, rapid analysis of macronutrients (including lactose, fat and protein) and milk energy in breast milk is of clinical importance. This study compared the macronutrients results of a mid-infrared (MIR) analyzer and an ultrasound-based breast milk analyzer and unified the results by machine learning.

**Methods:**

This cross-sectional study included breastfeeding mothers aged 22–40 enrolled between November 2019 and February 2021. Breast milk samples (n = 546) were collected from 244 mothers (from Day 1 to Day 1086 postpartum). A MIR milk analyzer (BETTERREN Co., HMIR-05, SH, CHINA) and an ultrasonic milk analyzer (Honɡyanɡ Co,. HMA 3000, Hebei, CHINA) were used to determine the human milk macronutrient composition. A total of 465 samples completed the tests in both analyzers. The results of the ultrasonic method were mathematically converted using machine learning, while the Bland-Altman method was used to determine the limits of agreement (LOA) between the adjusted results of the ultrasonic method and MIR results.

**Results:**

The MIR and ultrasonic milk analyzer results were significantly different. The protein, fat, and energy determined using the MIR method were higher than those determined by the ultrasonic method, while lactose determined by the MIR method were lower (all *p* < 0.05). The consistency between the measured MIR and the adjusted ultrasound values was evaluated using the Bland-Altman analysis and the scatter diagram was generated to calculate the 95% LOA. After adjustments, 93.96% protein points (436 out of 465), 94.41% fat points (439 out of 465), 95.91% lactose points (446 out of 465) and 94.62% energy points (440 out of 465) were within the LOA range. The 95% LOA of protein, fat, lactose and energy were - 0.6 to 0.6 g/dl, -0.92 to 0.92 g/dl, -0.88 to 0.88 g/dl and - 40.2 to 40.4 kj/dl, respectively and clinically acceptable. The adjusted ultrasonic results were consistent with the MIR results, and LOA results were high (close to 95%).

**Conclusions:**

While the results of the breast milk rapid analyzers using the two methods varied significantly, they could still be considered comparable after data adjustments using linear regression algorithm in machine learning. Machine learning methods can play a role in data fitting using different analyzers.

## Background

Breast milk macronutrients, including fat, carbohydrates (mainly lactose) and protein provide indispensable benefits for the growth of newborns. The only source of nutrition in early infancy is breast milk, so the energy of breast milk is also crucial to the growth of infants. Due to individual differences, the nutrient composition of human breast milk (such as protein and fat) can sometimes be insufficient to meet the needs of infants with special health conditions (for example, premature infants) [1–3]. Thus, it has become a common practice in nutritional support for preterm infants to strengthen human milk nutrition [4, 5]. Detecting the macronutrient content of human milk and adjusting its quantity and fortification can make fortified breast milk more suitable for the individual needs of preterm infants [1–3]. The development of metabolomics has allowed the understanding of the composition and the dynamic changes of breast milk and metabolic pathways [6–10]. Numerous chemical methods have been established to determine fat, protein, and lactose in human milk, and new techniques have recently been developed and reviewed by various researchers [3, 11, 12]. However, most methods with high accuracies, such as mass spectrometry, are expensive and thus can be challenging to popularize.

Because of the need for quick and cost-friendly analysis in clinical use, mid-infrared (MIR) and ultrasound methods have been rapidly developed and applied in neonatal wards, neonatal intensive care units, and maternal and child health care institutions [13, 14]. MIR is a method based on infrared chromatography (IRS) and has been widely used in quickly analyzing human milk macronutrients [12, 13, 15–22]. The technology uses the different wavelengths of infrared energy absorption of protein, fat, and carbohydrate to determine the contents [20]. Another popular rapid analysis technique is the ultrasonic method, based on high-frequency ultrasound radiation passing through the sample material [23–26]. However, there have only been a few reports analyzing the accuracy of ultrasonic methods, and the accuracy of the ultrasonic method needs further research [15, 27].

Rapid analysis techniques of breast milk components have been found to be particularly useful in various clinical applications [11, 13, 14, 16, 28]. However, other experts hold contrasting views. One reason is that unifying the results obtained from different methods can be challenging [16, 17]. Previous studies have found that rapid milk analyzers may be used in clinical routine to measure milk macronutrient but will require major adjustments [16, 17]. Another study found that routine clinical use of bedside or rapid measurement of human milk contents still lacks evidence and should not be recommended [18].

Up to now, machine learning has numerous applications in nutrition (e.g., automatic diagnosis and model fitting) [29–33]. Machine learning, more specifically the field of predictive modeling is primarily concerned with minimizing the error of a model or making the most accurate predictions possible, at the expense of explainability [34]. In applied machine learning, algorithms from many different fields, including statistics are borrowed and reused [34]. Linear regression is one of the most well-known and well understood algorithms in statistics and machine learning. Linear regression was developed in the field of statistics and is studied as a model for understanding the relationship between input and output numerical variables, but has been borrowed by machine learning.

Therefore, this study aims to compare the results of the MIR and ultrasound methods in rapid milk analysis and provide alternative approaches for rapid breast milk detection. These goals were achieved using linear regression algorithm in machine learning.

## Materials and methods

### Objects of study

From November 2019 to February 2021, volunteers were recruited from the Shanghai Xinhua Hospital for the study. All recruits were Chinese women who had lived in Shanghai for at least six months.

#### Inclusion criteria

Chinese mothers who have been breastfeeding (including all lactation stages); living in Shanghai for more than six months [35].

#### Exclusion criteria

Lack of breast milk led to the cessation of breastfeeding; inability or unwillingness to provide milk; unable to communicate due to language barriers or mental problems; had severe medical condition(s) requiring medication; lost contact before collecting breast milk; failure to collect breast milk as required; failure to store breast milk as required [35].

After getting briefed on the screening and inclusion criteria, the volunteer mothers were asked to provide one or more breast milk samples at their discretion. Multiple milk samples were provided at intervals of more than one month. The milk sample collection was accomplished by each volunteer at home, and the samples were then transported to the Xinhua Hospital via cold chain express for treatment and storage.

Chinese mothers aged 22–40 years old and who have been breastfeeding were enrolled in the study. Breast milk samples (n = 546) were collected from 244 Chinese mothers (from Day 1 to Day 1086 postpartum). In total, 546 milk samples were analyzed using the MIR method, while 465 were evaluated using the ultrasonic method. Eighty-one samples were not able to undergo ultrasonic analysis because the sample volume was insufficient.

**Fig. 1:**
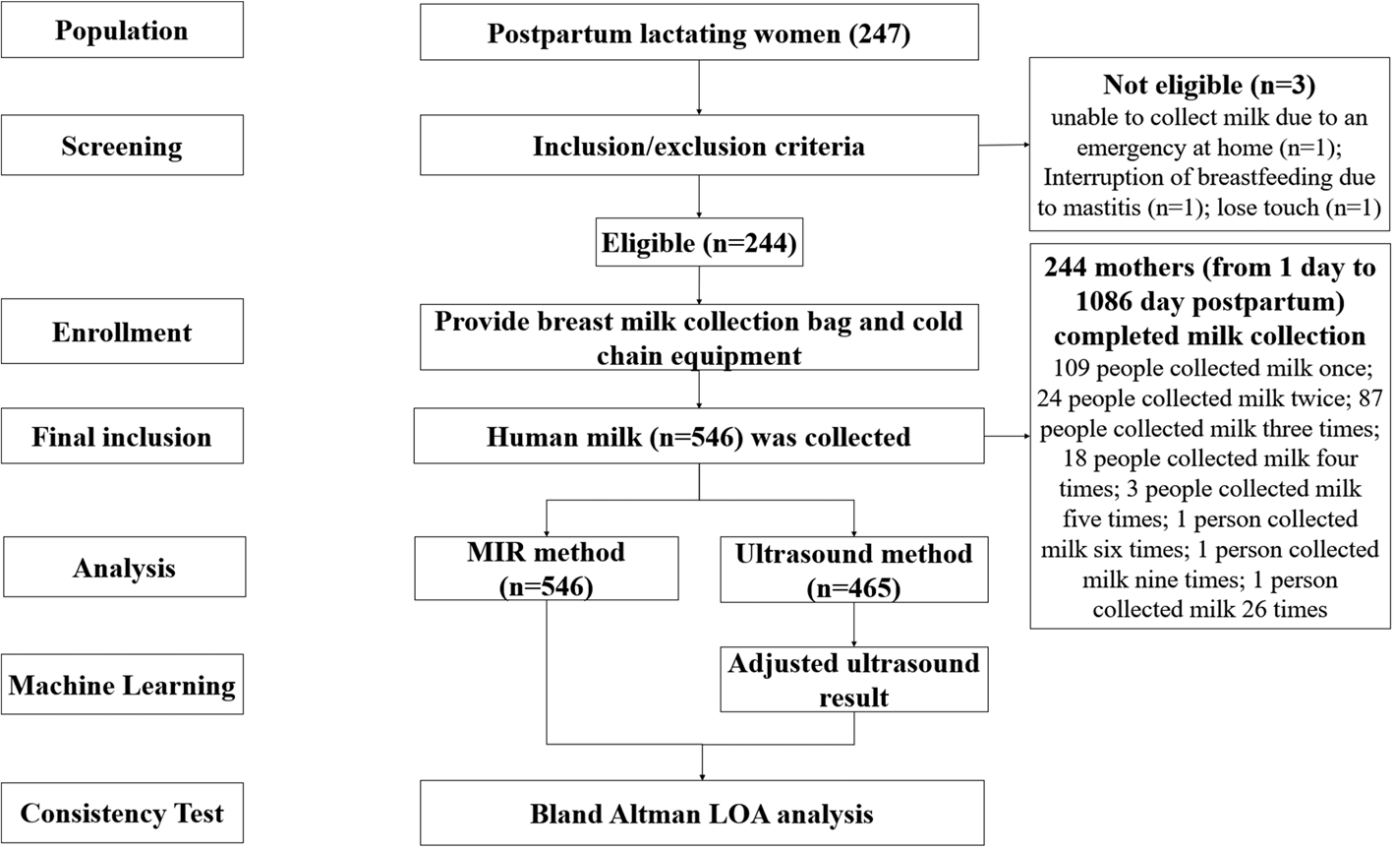
Study design flow chart. MIR: mid-infrared

 This study had been approved by the Ethics Committee of the Xinhua Hospital with approval number XHEC-C-2020-081, and each patient’s written informed consent was obtained prior to inclusion. The study design flow chart is shown in Fig. 1.

### Breast milk collection

The volunteers were first given a detailed explanation by face-to-face or via WeChat regarding the unified sample collection procedure. According to the collection protocol, the breast milk collection was to be completed at home, and the volunteer would have to collect the sample after fasting (not eating energy containing foods and drinks) for more than 8 h and before feeding the baby in the morning. Each volunteer would use an electric breast pump to suck the milk from one side of the breast until it was empty [15, 36]. The breast for milk collection was chosen at random by the volunteer. The following electric breast pump was recommended for milk sample collection, including Medela Co., Sonata Flex, USA; Medela Co., Freestyle Flex, Switzerland; Medela Co., Swing Maxi Flex, China; Medela Co., Swing maxi Flex, Switzerland; Medela Co., Swing Flex, Switzerland; Philips Co., Avent SCF303, China; Philips Co., Avent SCF316, China. The volunteer mothers must reconfirm with the researcher before collecting breast milk if the above model of breast pump wasn’t available. Manual breast pump was not allowed in this study. The mother would then have to homogenize the milk manually by carefully shaking the container in which the milk was pumped into, take 15ml of the mixture, and put it into the unified breast milk collection bag, stored at − 20 ℃. The remaining milk that was pumped was offered to the infant. The milk sample was then transferred via the cold chain within two days after collection.

After each sample was received, the milk properties were evaluated by the research group. If the samples were agglutinated, stratified, or had odor or other conditions, milk would have to be recollected within three days. Milk collection methods can be obtained from a previous literature [35]. Qualified samples were then manually homoginized by shaking the breast milk collection bag gently for 30 s until mix thoroughly and repacked [35]. All breast milk samples were divided into one 0.5 mL (for the MIR method) and 10ml vials (for the ultrasonic method) [17, 22]. The collected and encapsulated milk samples were stored in an exclusive freezer at − 80 ℃ until analysis [15, 37]. The researchers analyzed the collected samples every 60 days. The above process was performed for each breast milk collection.

### Milk sample treatment

Before analysis, the stored milk samples were put in a 37℃ water bath until it was completely dissolved, checked to ensure that the sample temperature can meet the working requirements of the analyzer (the MIR analyzer will automatically measure the temperature of samples before detection), and manually homogenized to ensure constant mass [17, 28]. Each sample was thoroughly mixed by turning the sample tube upside down gently for several times for 30s [17, 22]. To exclude human factors, all sample treatments prior to analysis were conducted by one researcher.

#### Sample analysis

In this study, two rapid breast milk analyzers based on different methodologies were used to detect milk samples repeatedly. One researcher conducted all sample analyses.

#### MIR milk analyzer

The MIR spectroscopy method is considered reliable and has been widely used in previous studies [14, 16, 19, 21, 22, 38, 39]. For this study, we used the MIR-based milk analyzer (BETTERREN Co., HMIR-05, SH, CHINA) to analyze milk composition (lactose, protein, fat, energy). This analyzer is an improved version of the classic instrument (MIRIS, Uppsala, Sweden) and was patented by the State Intellectual Property Office of the people’s Republic of China (No.: CN 108,760,671 A) in 2018.

According to manufacturer’s recommendations, the ambient temperature of the instrument should be maintained between 25 and 35 ℃. Before the daily sample testing, the built-in manufacturer’s quality control material was used for calibration. A special straw provided by the instrument supplier was used to take 0.5mL of homogenized milk sample, which was then transferred to a transparent covered cuvette, ensuring that there were no bubbles. The researcher handled the whole process carefully to avoid milk splashing or bubbles. The cuvette was then placed on the instrument and scanned for 10 s. The sample temperature detected by the analyzer should between 20 and 25 ℃. If it is lower or higher than the temperature range, the analyzer will warn and refuse to analyze the sample. Each milk sample had its ID number and was without sensitive information. In this study, we used raw data obtained by optical analysis without digital modeling (including lactation time), and the initial test results were stored in a password-protected database.

#### Digital ultrasound milk analyzer

Another milk analyzer (Honɡyanɡ Co,. HMA 3000, Hebei, CHINA) used in this study is based on the ultrasonic method. In one published article, a breast milk sample was assessed using an ultrasonic analyzer (the same brand as the present study) six times which resulted in a relative standard deviation (RSD) of 0.08–3.79% [26]. This suggests that the instrument has good reproducibility and high recovery rating (95–99%). The macronutrient content error using the digital ultrasound milk analyzer was less than 2% compared to the results from traditional methods [26]. The analyzer was stored in a cool and dry environment to avoid strong light and direct sunlight. The homogenized milk samples were placed on the test tube rack of the analyzer, and the test button was pressed, which started the testing process. The analyzer automatically moved the sample to the front of the detector near the sampling tube and inserted the sampling tube into the sampling tube at the bottom. The measurement time of each sample was about 20 s, and a maximum of 23 samples could be detected each round. After each analysis round, the analyzer will start the automatic flushing procedure. The results were automatically displayed on the computer connected to the analyzer when each sample test was completed. After completing the one-day test, the researcher used acid and alkaline cleaning solutions to complete the final cleaning procedure. The equipment manufacturer provided the neutral, acid, and alkaline cleaning solutions mentioned. The results were then stored in PDF format.

#### Statistical analysis

Statistical analysis was performed using SPSS Statistics 25.0 (IBM Co., Armonk, NY, USA). Continuous variables were presented as mean ± SD. The results between the two groups were compared using paired t-test. Machine learning algorithm used in the present study was linear regression, and the machine learning model’s performance was evaluated by variance score [17]. Python 3.6 (Python Software Foundation ) was used to train and test the data set for fat and energy, and the following python packages (NumPy 1.19.5; Matplotlib 3.3.4; Scikit-learn 0.24.2; Pandas 1.1.5; SciPy 1.5.4) were used. Excel (Microsoft®Excel®2016MSO) was used to develop the model for lactose and protein. Bland–Altman analysis was used to measure consistency between the measured MIR and the adjusted ultrasonic results using MedCalc 19.0.7 (MedCalc software Ltd, Ostend, Belgium). Bland–Altman scatters plot displayed the mean difference and limits of agreement (LOA). If the difference between the measurements (bias) was close to zero and the 95% LOA was within the clinically acceptable range, the measurements would be considered to have good consistency. A *p-value* less than 0.05 indicates statistically significant differences.

## Results

### Human milk composition of two analyzers

Table 1 shows the results of the samples measured by both MIR and ultrasound methods. Our results showed statistically significant differences between groups (all *p* < 0.001). After applying some adjustments, the ultrasound results were again compared with the MIR results using paired t-test (see Table 1). The results showed no significant difference between the adjusted ultrasound results and the MIR results (all *p* > 0.05).

**Table 1:** Main measurement results of two milk analyzers (mean ± SD)

	MIR (n = 546)	Ultrasound (*n* = 465)	Adjusted ultrasound (*n* = 465)	*P*-value^a^	*P*-value^b^
Protein (g/dl)	2.27 ± 0.20	1.29 ± 0.26	2.28 ± 0.26	< 0.0001	0.9998
Fat (g/dl)	3.09 ± 0.77	2.83 ± 1.18	3.15 ± 0.54	< 0.0001	0.8396
Lactose (g/dl)	6.03 ± 0.39	7.33 ± 0.34	5.99 ± 0.34	< 0.0001	0.9997
Energy (kj/dl)	268.51 ± 33.69	259.07 ± 46.48	269.80 ± 20.78	< 0.0001	0.9224

Paired t-test was used for comparison between groups; Continuous variables were presented as mean ± SD; MIR: mid-infrared

^a^ refers to the comparison between MIR and ultrasound results

^b^ refers to the comparison between MIR and adjusted ultrasound results

### Adjustment of the ultrasonic method results using machine learning

Since we found significant differences in the macronutrient analyses using different instrument, we used machine learning to generate mathematical models for three macronutrients and energy of human milk and converted the values of the ultrasonic measurements. The purpose of this step is to adjust the ultrasonic results and make them as close as possible to the MIR values.

**Fig. 2:**
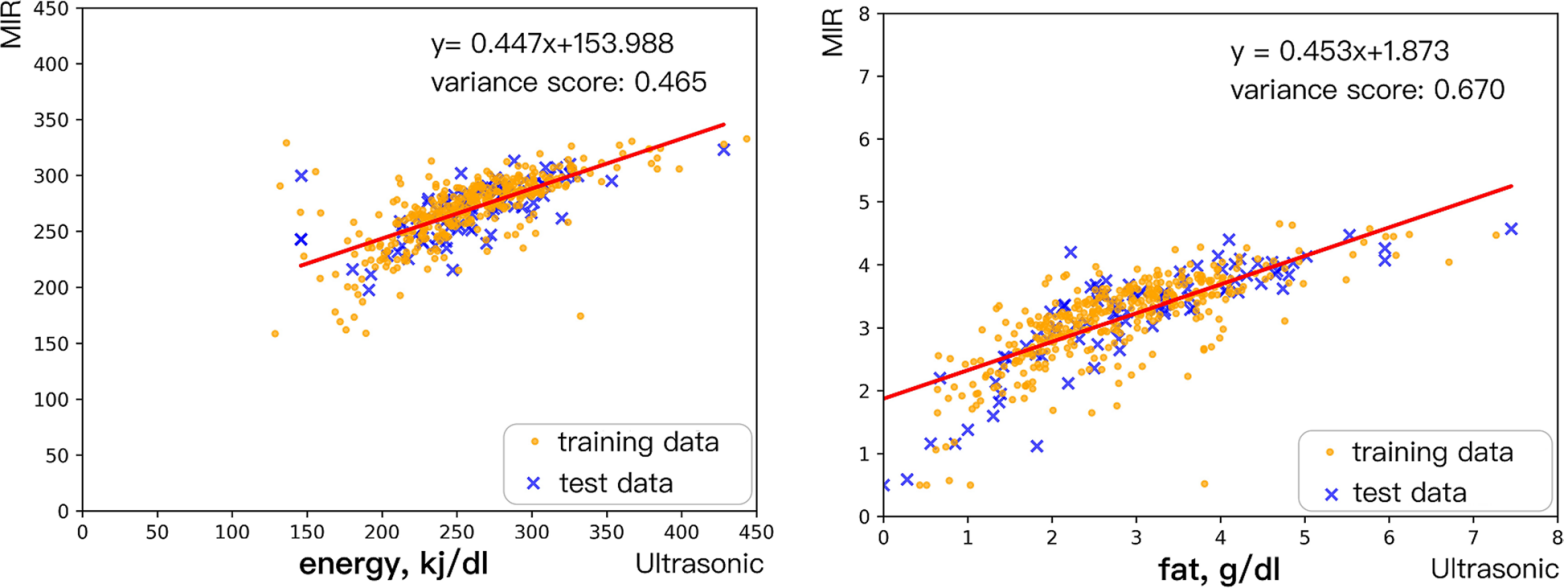
XY-plots for fat and energy. Y referred to the MIR method, x refers to the observed ultrasound method

The random algorithm was used to generate the data model between MIR and the initial ultrasound results. First, we established the data set according to Mersenne Twister(MT), which can quickly generate high-quality pseudo-random numbers and correct defects in the classical random number generation algorithm [40]. The machine learning model was produced by randomly sampling the data from the database. Training and test dataset were created by using the following code: klearn.model_selection.train_test_split (X, Y, test_size = 0.20, random_state = 1), X refers to ultrasonic, Y refers to MIR, and then sklearn.linear_model.LinearRegression (scikit-learn 0.24.2) was used for model training. We randomly selected 80% of the dataset to be used in generating the working model (called “training data”) and the other 20% to test the model (called “test data”), as recommended in previous literature [41–43]. These steps were used in creating the correction models for protein, fat, lactose, and energy content. Since the results for protein and lactose did not conform to the linear regression model after observation, they were translated using the mean value. The training data and the test data of fat and energy as well as the related statistical parameters are shown in Table 2. Figure 2 shows the training and test data and the linear equation for fat and energy.

**Table 2:** The training data and the test data of fat and energy of two milk analyzers (mean ± SD)

	Data set (*n* = 465)	MIR	*P*-value^a^	Ultrasound	*P*-value^a^	95% LOA^b^
Fat (g/dl)	**Training data (*n*** **= 372)**	3.14 ± 0.69	0.39	2.79 ± 1.14	0.13	-1.87 ~ 1.18
	**Test data (*n*** **= 93)**	3.21 ± 0.84		2.99 ± 1.32		-1.76 ~ 1.34
Energy (kj/dl)	**Training data (*n*** **= 372)**	269.62 ± 29.81	0.89	258.50 ± 47.08	0.60	-76.88 ~ 54.64
	**Test data (*n*** **= 93)**	270.07 ± 24.84		261.36 ± 43.64		-71.84 ~ 54.42

^a^ refers to the p-value of t-test between training dataset and test dataset; ^b^ refers to the result of Bland-Altman analysis between observed ultrasonic and MIR values

Finally, the linear regression equation (y = ax + b) between observed and adjusted ultrasound values was established, where y refers to the adjusted ultrasound values, and x refers to the observed ultrasound values.

**Fig. 3:**
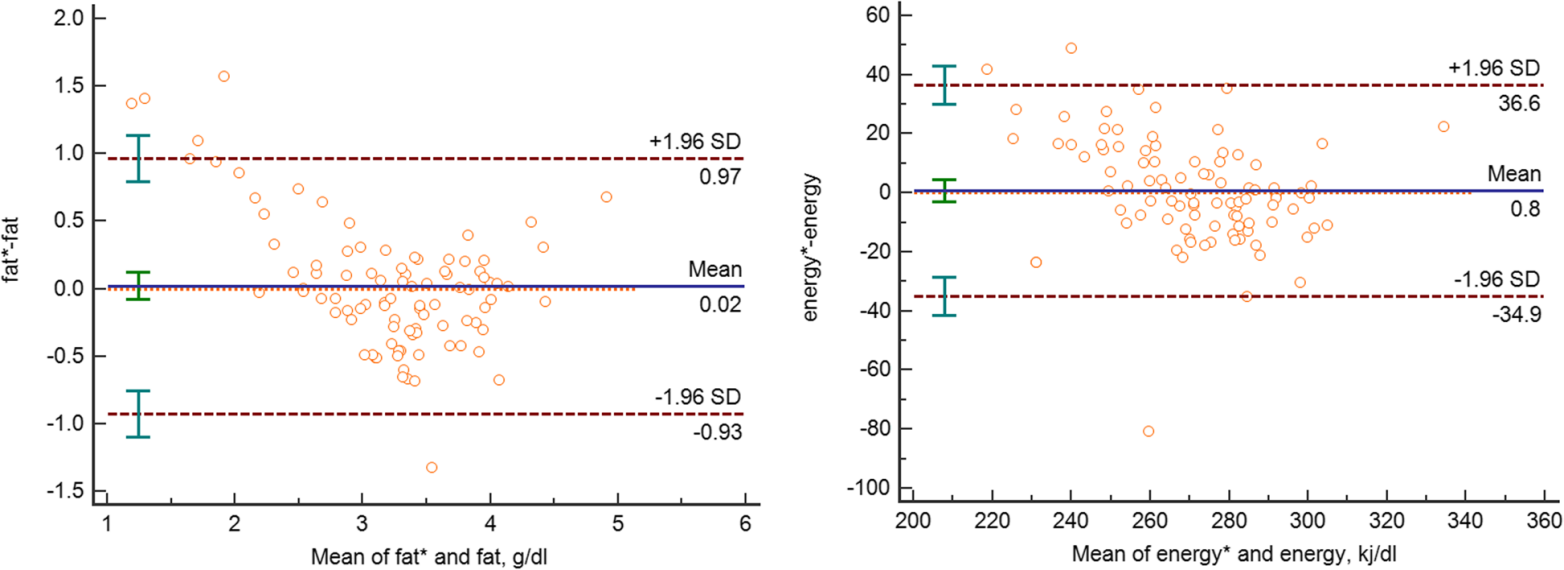
Bland–Altman scatter plots for fat, and energy of MIR and adjusted ultrasound values (test dataset).* refers to the adjusted results of the ultrasonic milk analyzer (test dataset)

We obtained four adjustment equations for protein, fat, lactose and energy:

- Adjusted fat: y = 0.453x + 1.873, mean squared error: 0.231, variance score: 0.670;
- Adjusted energy: y = 0.447x + 153.988, mean squared error: 329.751; variance score: 0.465;
- Adjusted protein: y = x + 0.98447; mean squared error: 0.095; variance score: not avalible;
- Adjusted lactose: y = x-1.34567; mean squared error: 0.201; variance score: not avalible;

The adjusted ultrasonic values were obtained using the above steps. The training data and the test data of the adjusted ultrasound results and the related model fitting parameters can be seen in Table 3. The consistency between the measured MIR (test dataset) and the adjusted ultrasound values (test dataset) was evaluated using the Bland-Altman analysis and the scatter diagram was generated to calculate the 95% LOA, as shown in Fig. 3. The 95%LOA of fat and energy were - 0.93 to 0.97 g/dl and - 34.9 to 36.6 kj/dl, respectively. The results suggest that after adjustments, the test data values generated by the two methods are more comparable than the results before adjustment (as shown in Tables 2 and 3).

**Table 3:** The training data and the test data of the adjusted ultrasound results and the model fitting parameters

	Data set (n = 465)	Adjusted ultrasound	95% LOA^a^	Mean squared error (MSE)	Variance score	Lin’s Concordance Correlation Coefficient	*P*-value^b^	Model MSE	Model Bias	Model Variance
Fat (g/dl)	**Training data (*n*** **= 372)**	3.14 ± 0.49	-0.91 ~ 0.91	0.217	0.551	0.67	0.13	0.233	0.231	0.002
	**Test data (*n*** **= 93)**	3.23 ± 0.57	-0.93 ~ 0.97	0.231	0.670	0.67				
Energy (kj/dl)	**Training data (*n*** **= 372)**	270.26 ± 20.71	-41.42 ~ 41.42	445.401	0.499	0.71	0.60	333.67	330.35	3.321
	**Test data (*n*** **= 93)**	271.52 ± 19.20	-34.92 ~ 36.58	329.751	0.465	0.78				

^a^ refers to the result of Bland-Altman analysis between adjusted ultrasonic and MIR values; ^b^ refers to the p-value of t-test between training data and test data

### The consistency and LOA of the MIR method and adjusted ultrasound values

The Bland Altman LOA analysis was proposed by Bland JM and Altman DG in 1986 to calculate the limits of agreement between two measurement results. Consistency is assessed by generating a scatter plot visualizing the deviation between each pair. If the values of one method are considerably greater (or less) than that of the other, the difference is called bias. Bias can be calculated using the mean difference between the two methods, and the variation is expressed by the standard deviation (SD) of the difference. If the difference follows a normal distribution, 95% of the points should be within the mean ± 1.96 SD. This interval is called the 95% LOA [44–46].

**Fig. 4:**
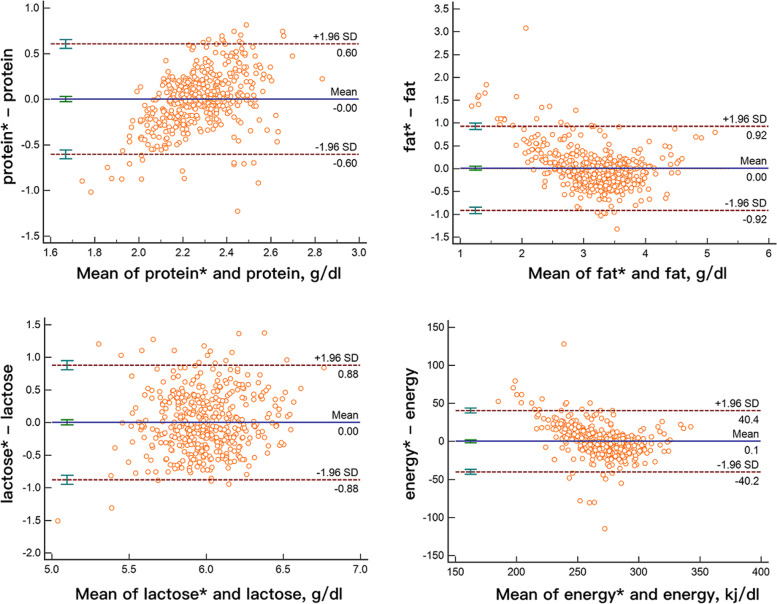
Bland–Altman scatter plots for protein, fat, lactose, and energy of MIR and adjusted ultrasound values. * refers to the adjusted results of the ultrasonic milk analyzer

After building the model, the consistency between the measured MIR and the adjusted ultrasound values was evaluated using the Bland-Altman analysis and the scatter diagram was generated to calculate the 95% LOA. After complete dataset adjustments, 93.96% protein points (436 out of 465), 94.41% fat points (439 out of 465), 95.91% lactose points (446 out of 465) and 94.62% energy points (440 out of 465) were within the LOA range. The 95% LOA of protein, fat, lactose and energy were - 0.6 to 0.6 g/dl, -0.92 to 0.92 g/dl, -0.88 to 0.88 g/dl and - 40.2 to 40.4 kj/dl, respectively, and clinically acceptable, as shown in Fig. 4. The results suggest that after adjustments, the values generated by the two methods are comparable.

## Discussion

We evaluated the difference between the measured values from milk analyzers based on the MIR and the ultrasound methods and used linear regression algorithm in machine learning to adjust the data and verify the consistency. The results of this study aid an understanding of the differences in measured values between popular milk analyzing approaches and can increased the practicability of commercially available rapid human milk analyzers.

The MIR analyzer is based on the principle of spectroscopy. Since the 1990s, the accuracy of the MIR analyzer has been continuously studied for different populations and their milk samples [11, 13, 17]. Compared with chemical approaches, the MIR method is often considered to have high accuracy in human milk composition analysis, although some studies have found that the results are not always accurate [12, 19, 39]. One study found that the accuracy of using MIR to determine protein and fat in human milk is about 60 − 70% [17]. Another study reported that the MIR method could underestimate protein content in human breast milk [47]. Other studies have shown that the MIR method could be used to determine the macronutrients in cow’s milk and predict other characteristics of dairy products (such as various fatty acids, milk protein composition and structure, curd characteristics, titrated acidity, and potential diseases) [48–51]. Digital modeling of the MIR analyzer is difficult, and the optical system is complex. This causes the overall price of MIR instruments to soar, limiting their use and access.

One popular alternative to the MIR is the ultrasonic analyzer. It uses digital ultrasonic technology to establish the mathematical model by measuring the sound velocity, attenuation, and impedance and the adiabatic compression coefficient of different human milk components to detect the milk’s protein, fat, lactose, minerals, water and energy contents [12, 27]. The main advantages of the ultrasonic instrument are that the equipment is cheap and easy to obtain and that the algorithm is simple. However, this approach has some major disadvantages, such as requiring more milk (5-10ml) than other methods, having longer testing time, and being easy to block. While the accuracies of these two methods still require further verification, considering that these two are the widely used methods for rapid breast milk analysis, it is necessary to use mathematical techniques to adjust the results to make them comparable.

In previous literature, the measurements of human breast milk from ultrasonic milk analyzer and MIR were found to be consistent [26]. However, in our study, the measured values using the two analyzers were not comparable. Although MIR and NIR methods have been used in clinical setting in some areas, some researchers have found that the results of existing milk analyzers require significant adjustments [17]. The main reasons include different methodologies and features, the absence of breast milk standards, and differences in sample preparation procedures [14, 19].

In this study, we used machine learning to convert the measured values of the ultrasonic method and verify the consistency. The results show that with the aid of machine learning, the adjusted ultrasound values could be consistent with the MIR results. Machine learning (linear regression algorithm) was used to establish the correction models for fat and energy. For protein and lactose, since the values did not conform to the linear regression model, they were converted based on the mean value. A possible reason for this is that other variables beyond the scope of this study could have affected the values for lactose and protein, such as maternal and infant information, details of breast milk (e.g., which breast the milk is collected from) and detection environment (e.g., temperature) [17, 25]. Another possible reason is that the measurement error of the instrument itself could have led to the above results. As for the fat and energy values obtained through the linear regression algorithm of machine learning, if more information can be included, the reliability of the model may be increased [34]. Future research can be carried out to develop more accurate models.

One major limitation of this study is its small sample size. The sample size was considerably affected by data availability, which could limit the applicability of the results. Another limitation is that the study focuses only on two milk analyzers, which could weaken the applicability of the research results. The selection of analyzers is another limitation; only two analyzers were used for comparison. Future studies may test other commercially available analyzers to better understand whether the model obtained by machine learning may be applicable. In addition, although the use of multiple samples from one mother for analysis and statistics has been widely used in previous studies, it is still necessary to consider the fact that the composition of human breast milk is highly variable inter- and intra-individually, which has not been fully considered in this study. The above deficiencies should be fully considered in future research.

## Conclusions

The present study evaluated the results of rapid milk analyzers based on MIR and ultrasonic methods. The results showed significant differences between the two methods in determining the macronutrients and energy in breast milk. We then used the linear regression algorithm in machine learning to adjust the measurements of the ultrasonic analyzer, and the adjusted results were consistent with the MIR results. The results suggest that machine learning can play a role in data fitting for different methodological analyzers.

## Data Availability

All data generated or analyzed during this study are included in this published article.

## Abbreviations

MIR: mid-infrared
IRS: infrared chromatography
LOA: limits of agreement
NIR: near-infrared
RSD: relative standard deviation
MT: Mersenne Twister
